# An efficient image encryption model based on 6D hyperchaotic system and symmetric matrix for color and gray images

**DOI:** 10.1016/j.heliyon.2024.e31618

**Published:** 2024-05-27

**Authors:** Anju Panwar, Geeta Biban, Renu Chugh, Asifa Tassaddiq, Rabab Alharbi

**Affiliations:** a Department of Mathematics, Maharshi Dayanand University, Rohtak 124001, India; b Department of Mathematics, Gurugram University, Gurugram 122003, India; c Department of Basic Sciences and Humanities, College of Computer and Information Sciences, Majmaah University, Al Majmaah 11952, Saudi Arabia; d Department of Mathematics, College of Science, Qassim University, Buraydah, 51452, Saudi Arabia

**Keywords:** 15 Bxx, 68 Uxx, Image encryption, Symmetric matrix, Hyperchaotic system, Algorithms

## Abstract

The security of images is one of the predominant pivotal aspects in the mammoth and still expanding digital domain. Due to chaotic system properties i.e. randomness and unpredictability is very appropriate to encrypt the images. In this research article, we construct an encryption model via 6D hyperchaotic map and a symmetric matrix for both color and grayscale images. We utilize the 6D hyperchaotic map in the confusion stage to change the pixel location and the symmetric matrix is used for changing the pixel value in the diffusion step for each RGB channel extraction from plain or original image. The image encryption model is checked over differential attacks (NPCR and UACI). Histogram analysis, correlation coefficients, and entropy analysis are also performed as statistical attacks. In conclusion, the image pixels are uniformly distributed, and the average entropy value are 7.9992 and 7.9973 for color and grayscale images, subsequently. The average NPCR and UACI for color images are 99.5956 and 33.4061, correspondingly, while the values for grayscale images are 99.5934 and 33.3054, respectively. These values are in the vicinity of optimal ranges. The suggested scheme's great efficiency and the proposed algorithm's resilience to a wide range of cryptanalytic attacks are implied by experimental results, statistical analysis, and differential attacks.

## 1 Introduction

In digital multimedia communication, colored images convey personal information, banking details, national or international security documentation, and other exposure and gray images show sealed X-ray medical reports and it's also very useful for processing of segmentation. To protect images from intrusions and attackers, it is essential to adopt highly secure encryption techniques, making them acceptable for use in practical applications.

Chaos theory is an integrative field of scientific study summarized by American mathematician Edward Norton Lorenz. Chaos theory uses several kinds of chaotic maps that have inherent qualities like randomness, unpredictability, sensitivity to starting conditions, complicated behavior, control parameters, etc. that fulfill the rudimentary needs of image encryption. Two distinct forms of chaotic systems are used to create encryption algorithms: low-dimensional chaotic models, especially chaotic systems, and high-dimensional chaotic maps, particularly hyperchaotic models. In contrast to high-dimensional systems, low-dimensional maps are straightforward to apply, have a limited keyspace, and offer less protection. Due to two or more positive Lyapunov exponents, hyperchaotic systems exhibit more complex behavior than low-dimensional systems, resulting in broad key sequences and good security for encrypting the picture data.

Hosny et al. [1] constructed a cipher model follows two key steps: shuffling the pixels using the block scrambling technique and diffusing the image pixels utilizing the logistic map for color image. Teng et al. [2] used logistic and sine maps to produce a 2D hyperchaotic map and an encryption cryptography method for gray images. Erkan et al. [3] utilized a 2D Schaffer hyperchaotic model and constructed a color image cipher and decryption model where they performed permutation and diffusion steps. Zhang et al. [4] developed image cipher method via 2D sine-cosine coupling system. Jara et al. [5] defined the color image cipher model via elliptic curve cryptography, DNA encoding, and 4D hyperchaotic system. Wang et al. [6] used 4D chaotic system and a DNA sequence to construct the encryption scheme for color images. Nazir et al. [7] used 4D hyperchaotic model and genetic codes for cipher the image. Yan et al. [8] investigated an image cipher process that depends on a 4D hyperchaotic model for color image. By combining a DNA sequence with a new 5D hyperchaotic system, Li et al. [9] designed the frame for picture encryption. Qiu et al. [10] established a 2D hyperchaotic model (2D-CSCM) and pragmatic it to color image encryption for scrambling the image pixels using Rubik's cube. Mir et al. [11] used a chaotic system and an RSA cryptosystem to cipher color pictures. Wang et al. [12] generated an picture cipher technique for color images utilizing an improved quantum revolving gate and hyperchaotic system. Kumar et al. [13] encoded the color pictures via hybrid chaotic map. Li et al. [14] took a tent chaotic model to develop the cipher model. Enayatifar et al. [15] generated an picture cipher scheme based on a synchronous permutation approach to diffuse the images. A hyperchaotic map and the Fibonacci Q-matrix were utilized by Hosny et al. [16] to construct a framework for image encoded. For additional details on related topics, consider, [17], [18], [19], [20], [21], [22], [23], [24], [25], [26], [27], [28], [29], [30].

From the above literature review, notice that most of the encryption techniques depend on low dimensional chaotic maps or hyperchaotic maps containing low keyspace and are implemented only on gray or color images that motivate to design of the image cipher model via a high dimensional hyperchaotic model i.e. 6D hyperchaotic system that contains large key space for both color and gray images. This study aims to develop an encryption technique that is more effective and secure for color and grayscale images.

In public communication applications, the suggested image encryption and decryption system is applied to protect sensitive image information from malicious access. A digital color image is expressed by a three-dimensional matrix, where one dimension is allocated to each of the RGB. So, the picture is fragmented into color channels: red, green, and blue. The two main steps of image encryption methods are confusion (or scrambling) and diffusion. Because hyperchaotic systems offer more secure encryption algorithms, we chose the 6d hyperchaotic system in this research to confuse the pixels of the images for each RGB channel. To change the pixel value we are using a 2×2 symmetric matrix that gives more protection as compared to the real value, we adjust the pixel value for each RGB channel throughout the diffusion step.

The following provides an overview of the paper's principal features and achievements.

• We create a cryptographic model for encrypting and decrypting color and grayscale images that depend on a symmetric matrix and a 6D hyperchaotic system.
• We experiment image encryption algorithm on various 256×256 color and gray images especially “Pepper”, and “Baboon”.
• The histogram is utilized for examine the pixel's values distribution for each channel (RGB) in plain, cipher, and decrypted color images.
• While evaluating two consecutive pixels in plain or encrypted images, we used correlation coefficient analysis to determine how strongly they are correlated.
• Entropy analysis is used to verify the developed technique's safety, and it is contrasted with other encrypted algorithms for both color and grayscale images.
• Differential attacks are very helpful to protect the images from intrusions. We find out NPCR and UACI values for encrypted pictures and also checked against existing image encryption algorithms.

Most image cipher models are follows low-dimensional chaotic system and give less image protection from attackers. The novelty of the proposed image cipher technique is to protect the images from intrusions with the unpredictable key with large key space depending on the 6D hyperchaotic map and symmetric matrix.

This research article is classified as follows: Section 2 gives the preliminaries and frames out the encryption and decryption algorithm in Section 3. The encryption scheme for color and gray images leading to the experimental results is contained in Section 4. Section 5 composed examination of the algorithm and several outcomes based on statistical analysis are illustrated. Section 6 contained the comparison analysis of differential attacks i.e. NPCR and UACI. Finally, Section 6 provides a succinct summary of the research.

## 2 Preliminaries

### 2.1 6D hyperchaotic system [31]

The six-dimensional (6D) hyperchaotic system with quadratic nonlinearity is given as:

(1) $$\left\{ \begin{array}{c} \overset{˙}{x_{1}}=\tau_{1}(x_{2}-x_{1})+x_{4};\\ \overset{˙}{x_{2}}=\tau_{2}x_{1}-x_{1}x_{3}+x_{4};\\ \overset{˙}{x_{3}}=x_{1}x_{2}-x_{3}-x_{4};\\ \overset{˙}{x_{4}}=-\tau_{3}(x_{1}+x_{2)}+x_{5};\\ \overset{˙}{x_{5}}=-x_{2}-\tau_{4}x_{5}+x_{6};\\ \overset{˙}{x_{6}}=-\tau_{5}(x_{1}+x_{5}), \end{array}\right.$$

where constants $\tau_{1}$, $\tau_{2}$, $\tau_{3}$, $\tau_{4}$, and $\tau_{5}$ dictate the periodic, chaotic, hyperchaotic, and bifurcation behavior of the map. With $\tau_{1}=10,\tau_{2}=76,\tau_{3}=3,\tau_{4}=0.2,\tau_{5}=0.1$ for the starting condition (1,1,1,1,0,0), the 6D system produces hyperchaotic behavior. The following Lyapunov exponents (LE) describe the hyperchaotic system:

$LE_{1}=1.4620,LE_{2}=0.1433,LE_{3}=0.0725,LE_{4}=0.0449,LE_{5}=0,LE_{6}=-12.0700$.

A phase plot is a way to visualize dynamic systems with complex movements. 2D phase plots and 3D phase plots completely describe the trajectory of the 6D hyperchaotic system. Phase plots are very useful to see the motion of variables in the 6D hyperchaotic map.

**Figure 1:**
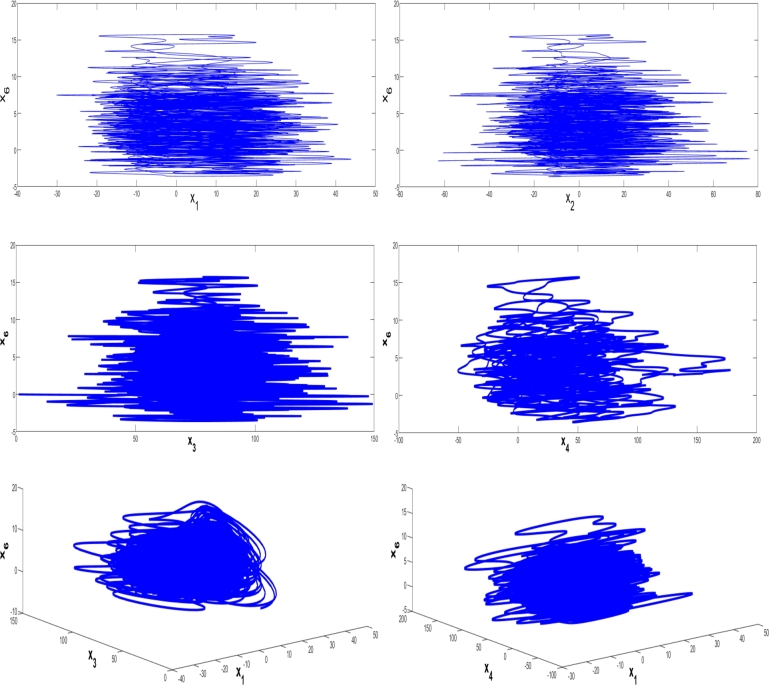
The 6D hyperchaotic system's MATLAB 2D phase plots and 3D phase plots for *X*(0)=(1,1,1,1,0,0) and (*τ*_1_,*τ*_2_,*τ*_3_,*τ*_4_,*τ*_5_)=(10,76,3,0.2,0.1).

Fig. 1 illustrates the 2 and 3-dimensional phase plots using the 6D hyperchaotic map X(0)=(1,1,1,1,0,0). The mapping $\left(\tau_{1},\tau_{2},\tau_{3},\tau_{4},\tau_{5})=(10,76,3,0.2,0.1\right)$ helps comprehend the 6D hyperchaotic map.

**Figure 2:**
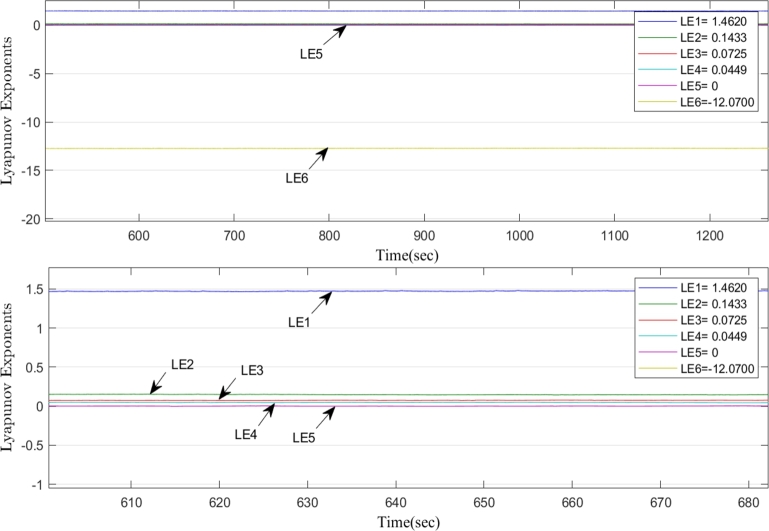
The 6D hyperchaotic system's LE's for *X*(0)=(1,1,1,1,0,0) and (*τ*_1_,*τ*_2_,*τ*_3_,*τ*_4_,*τ*_5_)=(10,76,3,0.2,0.1).

Fig. 2 depicts the map, which has four positive Lyapunov exponents, as being hyperchaotic.

We use 6D hyperchaotic map in the confusion step to change the location of the color and gray image pixels.

### 2.2 Symmetric matrix [32]

A square matrix equivalent to the transposition of itself is called a symmetric matrix. If *S* is a symmetric matrix, then it holds the condition: $S=S^{T}$, where $S^{T}$ is the transpose of the matrix *S*. This can be represented as: If $S={\left[s_{ij}\right]}_{n\times n}$ is the symmetric matrix, then $\left[s_{ij}]=[s_{ji}\right]$ for all i,j, where 1≤i,j≤n. Here, any natural number *n*, $s_{ij}$ is an element at location (i,j) where *i*th row and *j*th column in matrix *S*.

Symmetric matrix properties

• The difference and sum of two symmetric matrices is a symmetric matrix.
• If *S* is symmetric matrix then $S^{m}$ is symmetric matrix, where *m* is any integer.
• If *S* is a symmetric matrix as well as invertible, then the inverse of *S* i.e. $S^{-1}$ will also be a symmetric matrix.

We use a 2×2 symmetric matrix to encrypt the images and the inverse of the symmetric matrix is used for the diffusion step in the decryption process.

## 3 Algorithm

### 3.1 Encryption algorithm

In the cipher process, the plain color picture *D* is separated into three channels in the cipher process: *RGB*, and each channel is separated into blocks and sub-images. Algorithms for encrypting the images are based on two essential steps. The 6D hyperchaotic map is used in the confusion stage to change the pixel layouts using equation (1). Diffusion is the second stage, where the symmetric matrix modifies the pixel's value. The encryption technique's methodology is as follows:

**Step 1:** Separate given color picture *D* with size M×N into three gray images for *RGB* channels and transform picture *D* to *S* vector for each individual channel.

**Step 2:** The 6D hyperchaotic map is generated in the confusion stage. Using the system's starting condition, we determine

(2) $$x(1)=\frac{\sum_{i=1}^{MN}S(i)+(M\times N)(M\times N)}{9^{55}+(M\times N)}.$$

For computing the other initials, we use pseudocode:

“for i=2:6

x(i)=mod(x(i−1)⁎1e6,1)

end”,

where $1e6=1⁎10^{6}$, which is based on the image for each individual channel.

**Step 3:** Generated the 6D hyperchaotic system utilizing following pseudocode:

“τ(1)=10;τ(2)=76;τ(3)=3;τ(4)=0.2;τ(5)=0.1;

K=@(t,x(1))[τ(1)⁎(x(2)−x(1))+x(4)

τ(2)⁎x(4)−x(1)⁎x(3)+x(1)

x(1)⁎x(4)−x(3)−x(2)

−τ(3)⁎(x(5)+x(2))+x(1)

−x(2)−τ(4)⁎x(4)+x(6)

−τ(5)⁎(x(1)+x(5))];”

With the support of this approach, we created a new vector *K* and selected three sequences $(x_{1},x_{2},x_{6})K$ with *K* has a size M×N.

**Step 4:** Sequence *K* is sorting in ascending order to get the vector *L*, and then find $P=S(L_{i})$ permuted vector depending on *K*.

**Step 5:** We are using a symmetric matrix in the diffusion step. In this disorganized image i.e. the image gets after the confusion step, *P* is transformed into a matrix and divided into 2×2 sub-blocks.

**Figure 3:**
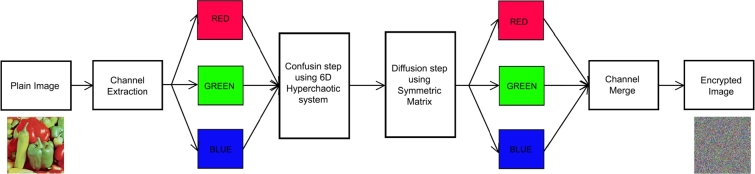
The encryption algorithm's block diagram.

**Step 6:** Each subblock is multiplied by the symmetric matrix $T=\left[\begin{array}{cc} 10 & 7\\ 7 & 5 \end{array}\right]$, and the result is the matrix *C* with the plain image D=C. Obtain the result as an encoded image *C*. Fig. 3 shows the suggested encryption algorithm's block diagram.

### 3.2 Decryption algorithm

The countermand encryption technique is carried through in this phase. The decryption procedure is carried out using the 6D hyperchaotic map. The *RGB* image channels of the color-encrypted picture are segregated. The decryption algorithm's methodology is as follows:

**Step 1:** Retrieve the ciphered image *C* and divide it into 2×2 subblocks for each *RGB* channel. To find scrambled image $K^{\prime }$, we apply the following diffusion equation using $T^{-1}$ on image blocks.

(3) $$\left[\begin{array}{cc} K_{m,n}^{\prime } & K_{m,n+1}^{\prime }\\ K_{m+1,n}^{\prime } & K_{m+1,n+1}^{\prime } \end{array}\right]=\left[\begin{array}{cc} C_{m,n} & C_{m,n+1}\\ C_{m+1,n} & C_{m+1,n+1} \end{array}\right]\left[\begin{array}{cc} 5 & -7\\ -7 & 10 \end{array}\right]mod\;256,$$

with n=1:3:5:...:N; m=1:3:5:...:M.

**Step 2:** Transform each channel's individual picture $K^{\prime }$ into *W* as vector.

**Step 3:** The following equation, which takes the generated vector *L*, allows each pixel to be returned to its initial location:

(4) $$Q(L_{i})=W_{i};i=1:MN.$$

**Figure 4:**
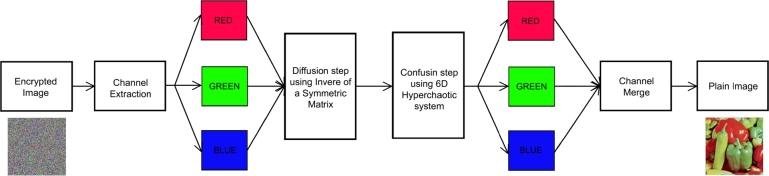
The decryption algorithm's block diagram.

**Step 4:** Transmogrify the *Q* into matrix *D* for each channel and C=D and receive the decrypted picture *D*. The proposed decryption algorithm's block diagram is manifest in Fig. 4.

## 4 Experimental results

**Figure 5:**
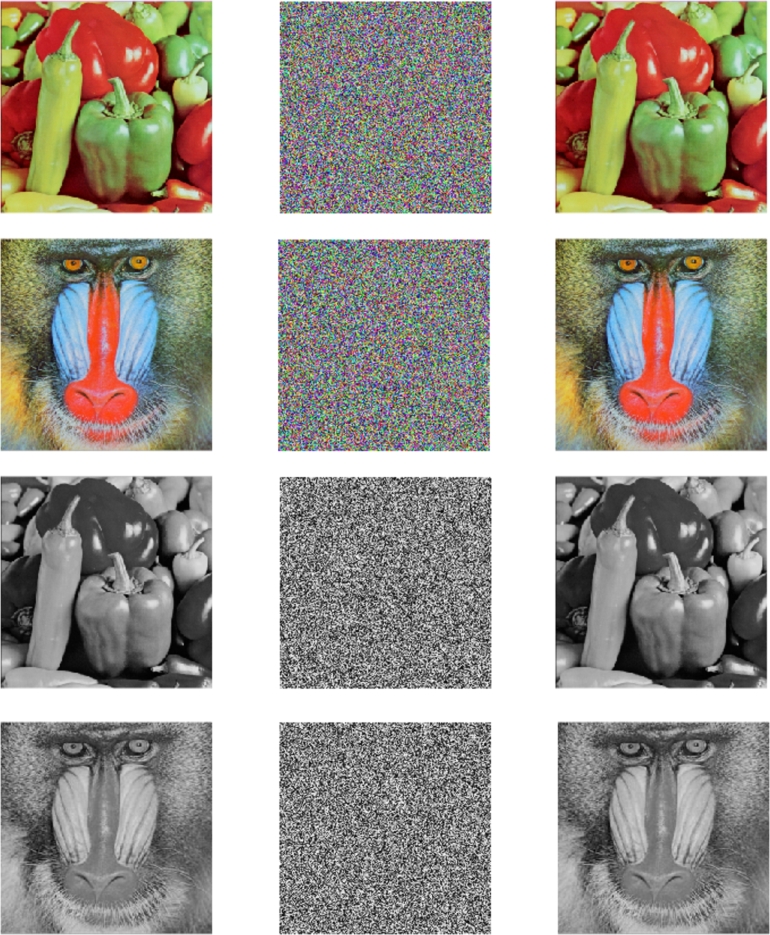
(a) Plain image (b) Cipher image (c) Decrypted image.

For the research, various 256×256 color and grayscale images, such as “Pepper,” and “Baboon,” were used. Fig. 5 represents (a) the plain image, (b) the corresponding encoded image, and(c) decrypted image.

The developed encryption and decryption procedure for color and gray images was performed utilizing Matlab 9.10.0 (R2021a), used in a personal computer along specifications with Microsoft Windows 11 version 21 H2 as the operating system, and hardware such as a CPU 1.60 GHz, Intel Core i5, 1 TB hard storage capacity, and 256 GB SSD.

## 5 Statistical analysis

The histogram, correlation, and entropy for the images are three climacteric statistical parameters of image encryption techniques. The proposed method's outcomes were contrasted with previous image encryption techniques.

### 5.1 Histogram analysis

**Figure 6:**
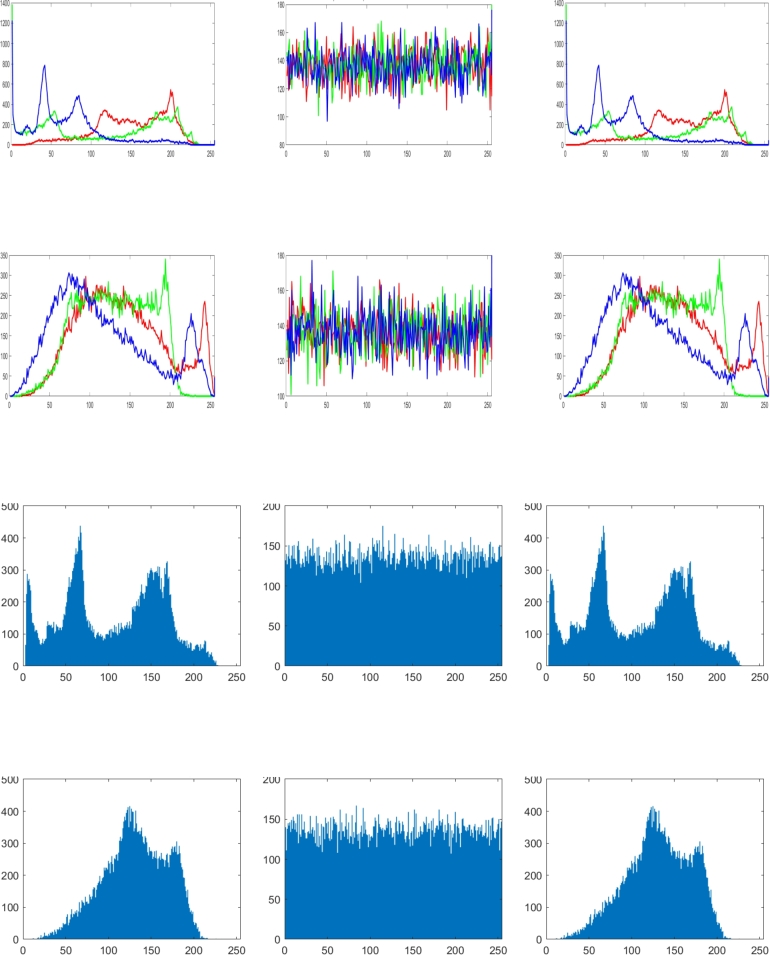
Histogram analysis in accordance with Fig. 5 for (a) Plain image (b) Cipher image (c) Decrypted image.

The original image's channel-by-channel value distribution can be visually represented using a histogram. Using histogram analysis, the quantifiable similarities between the plain and cipher pictures are identified. The basic image's histogram manages an arbitrary arrangement of pixels for each channel. The ciphered picture histogram, which means that neighboring pixels per RGB channel are uniformly distributed, provides unquestionable evidence that the presented method is robust to statistical attacks. We can observe from the outcome in Fig. 6, that the cipher image's RGB and grayscale pixel value distribution is uniform and the expected results are accomplished.

### 5.2 Correlation coefficient analysis

The degree of correlation between two adjacent pixels is estimated by the correlation coefficient. Using three neighborhood pixel directions, the correlation coefficient is determined as follows:

(5) $$r_{k,l}=\frac{E[(l-E(l))(k-E(k))]}{\sqrt{D(l)}\times \sqrt{D(k)}},$$

(6) $$E(k)=\frac{1}{T}\sum_{i=1}^{T}k_{i},$$

(7) $$D(k)=\frac{1}{T}\sum_{i=1}^{T}{\left(k_{i}-E(k)\right)}^{2},$$

where *T* denote the total adjacent pixels, and E(k) stand for the expectation of *k* and D(k) represents variance of *k*. The values of the image's extremely near pixels are $k_{i}$ and $l_{i}$.

**Figure 7:**
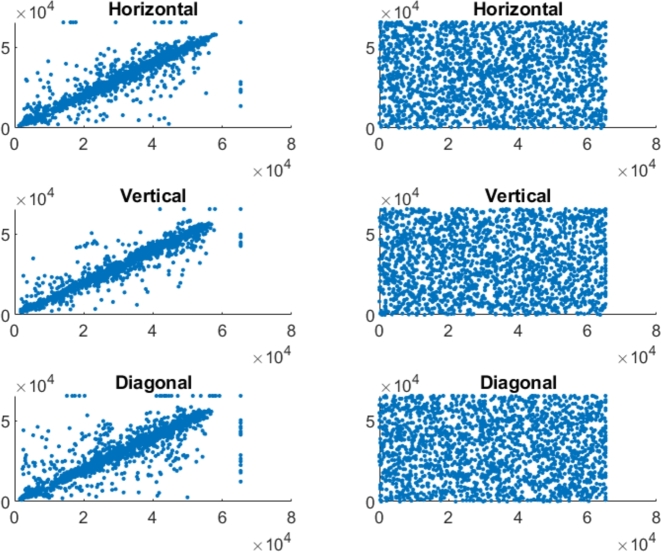
Adjacent pixel correlation of the color pepper image and its corresponding encrypted image.

**Figure 8:**
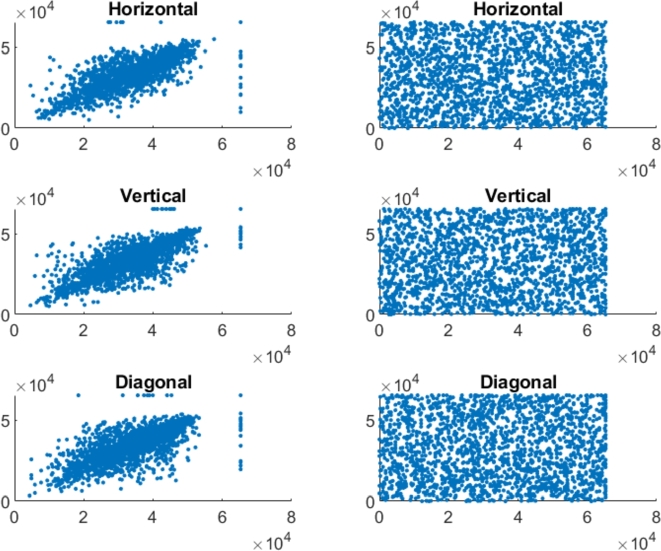
Adjacent pixel correlation of the color baboon image and its corresponding encrypted image.

**Figure 9:**
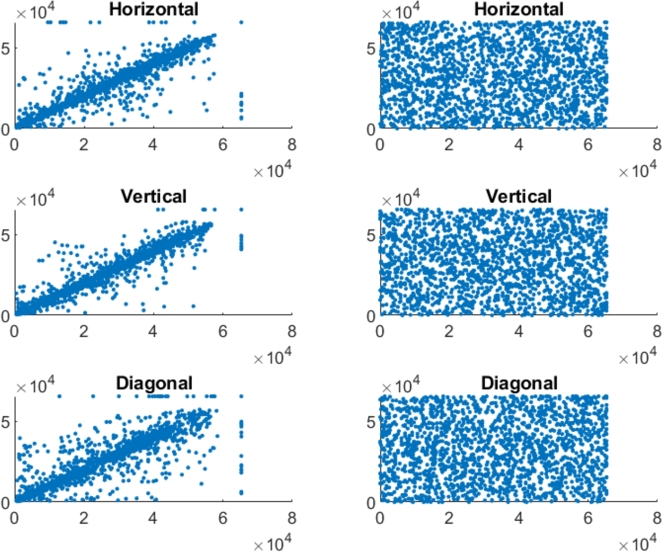
Adjacent pixel correlation of the gray pepper image and its corresponding encrypted image.

**Figure 10:**
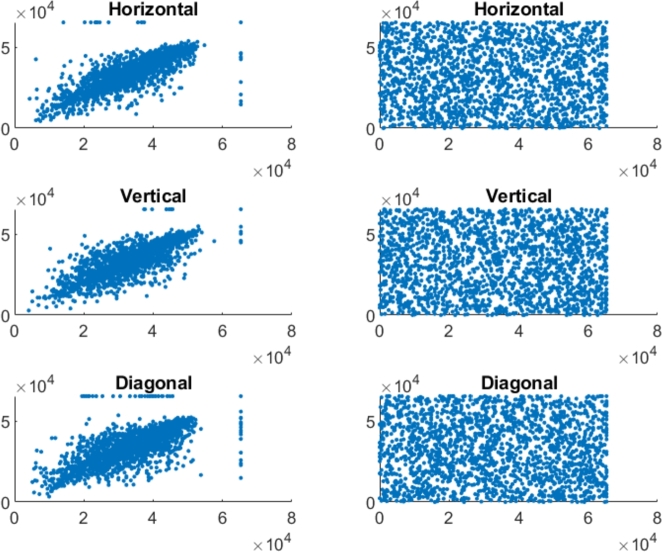
Adjacent pixel correlation of the gray baboon image and its corresponding encrypted image.

In the securely encrypted photos, there should be almost no correlation between neighboring pixels. Finding indicates that the suggested approach is able to remove the correlation between pixels in the encoded image. Figure 7, Figure 8, Figure 9, Figure 10 represents the correlation analysis for experimental images. In Figure 7, Figure 8, Figure 9, Figure 10 *X*-axis represents the pixel value for location (x,y), and the *Y*-axis shows the pixel value for location (x+1,y), (x,y+1), (x+1,y+1) for horizontal, Vertical, diagonal direction, respectively. Figures show the uniform distribution of surrounding pixels in the resultant images, indicating no correlation between neighboring pixels. Table 1, Table 2 depict the correlation coefficients for the various color and grayscale original pictures and their encrypted pictures that encrypted by numerous techniques.

**Table 1:** Comparison of the correlation coefficient between the suggested algorithm and alternative encryption methods for color images in the three orientations of H, V, and D.

				Average
Original Image	H	0.9709	0.9014	0.9361
	V	0.9784	0.8767	0.9276
	D	0.9531	0.8497	0.9014
Suggested	H	0.0018	0.0022	0.0020
	V	0.0004	0.0004	0.0004
	D	0.0006	0.0019	0.0013
Mir et al. [11]	H	0.0040	-0.0039	0.0000
	V	-0.0025	0.0003	-0.0011
	D	-0.0011	0.0026	0.0008
Wang et al. [12]	H	-0.0023	0.0032	0.0000
	V	0.0037	-0.0042	-0.0003
	D	0.0040	-0.0004	0.0018
Kumar et al. [13]	H	0.0045	−0.0145	0.0023
	V	−0.0021	0.0038	0.0019
	D	0.0075	0.0051	0.0063

**Table 2:** Comparison of the correlation coefficient between the suggested algorithm and alternative encryption methods for grayscale images in the three orientations of H, V, and D.

				Average
Original Image	H	0.9625	0.8826	0.9226
	V	0.9725	0.8377	0.9051
	D	0.9393	0.7902	0.8648
Suggested	H	0.0055	0.0069	0.0062
	V	0.0026	0.0017	0.0022
	D	0.0019	0.0053	0.0036
Li et al. [14]	H	0.0021	0.0055	0.0038
	V	0.0218	0.0015	0.0117
	D	0.0096	0.0041	0.0069
Enayatifar et al. [15]	H	0.0037	0.0059	0.0048
	V	0.0258	0.0041	0.0150
	D	0.0079	0.0028	0.0054
Hosny et al. [16]	H	0.0211	0.0065	0.0138
	V	0.0129	0.0337	0.0233
	D	0.0013	0.0244	0.0129

### 5.3 Entropy

Information entropy [33] is a magisterial concept in information theory that was first introduced by Claude E. Shannon. Entropy analysis is a crucial step in understanding the randomness and irrationality of images. The amount of randomness in the image is assessed using entropy. Entropy measures how evenly the image's gray values are distributed. As expressed in the equation of entropy:

(8) $$H(g)=\sum_{i=1}^{2^{w}-1}P(g_{i})\log_{2}\frac{1}{P(g_{i})},$$

where $P(g_{i})$ is occurrence probability of $g_{i}$, $2^{w}$ shows total quantity of $g_{i}$, and *w* is the total number of pixels in the picture. The absolute entropy value should be eight for good encrypted images. The information entropy values of the various color and grayscale images, as well as the respective encoded pictures by various models, are shown in Table 3, Table 4. The outcome shows that the majority of information entropy values of the developed methodology are appreciably near to the optimum information entropy in terms of mathematics.

**Table 3:** Comparison of entropy between several encryption systems and the suggested method for color images.

			Average
Original Image	7.7039	7.6868	7.6954
Suggested	7.9992	7.9992	7.9992
Mir et al. [11]	7.9963	7.9955	7.9959
Wang et al. [12]	7.9966	7.9971	7.9968
Kumar et al. [13]	7.9982	7.9978	7.9980

**Table 4:** Comparison of entropy between several encryption systems and the suggested method for gray images.

			Average
Original Image	7.5596	7.2297	7.3947
Suggested	7.9973	7.9973	7.9973
Li et al. [14]	7.9909	7.9912	7.9911
Enayatifar et al. [15]	7.9958	7.9938	7.9948
Hosny et al. [16]	7.9972	7.9975	7.9974

## 6 Differential attacks

These attacks consist of the unified average changing intensity and the number of pixel change rates i-e (UACI & NPCR). The number of pixels that have exchanged in two pictures has been ascertained using NPCR. *F* is shown as the image that's encrypted from the original picture and the picture is encoded from a plain picture with one and only one bit distinct is denoted by $F^{\prime }$. Assume that the values F(k,l) and $F^{\prime }(k,l)$ represent the pixels located at $k^{th}$ row and $l^{th}$ column of the respective images *F* and $F^{\prime }$. *P* shows the total pixels. NPCR as follows:

(9) $$NPCR=\frac{\sum_{k,l}E(k,l)}{P}\times 100,$$

where,

(10) $$E(k,l)=\{\begin{array}{cc} 0\; & \text{if }F(k,l)=F^{\prime }(k,l),\\ 1\; & \text{if }F(k,l)\neq F^{\prime }(k,l). \end{array}$$

Utilizing UACI, determine the average intensities difference between the two pictures, represented as follows:

(11) $$UACI=\frac{1}{P}\sum_{k,l}\frac{\left|F(k,l)-F^{\prime }(k,l)\right|}{255}\times 100.$$

The ciphered method works effectively if the NPCR and UACI values are closest to the optimum values, 33.4635 and 99.6094, respectively. Table 5, Table 6 represent the juxtaposition of NPCR and UACI for the suggested and already established schemes for color and gray images, respectively.

**Table 5:** Comparison of NPCR, UACI between several encryption systems and the suggested method for color images.

				Average
Suggested	NPCR	99.5901	99.6012	99.5956
	UACI	33.4166	33.3955	33.4061
Mir et al. [11]	NPCR	100	100	100
	UACI	33.30	33.48	33.31
Wang et al. [12]	NPCR	99.5422	99.6225	99.5607
	UACI	33.4614	33.532	33.4872
Kumar et al. [13]	NPCR	99.5952	99.5979	99.5960
	UACI	30.7212	29.6780	30.3768

**Table 6:** Comparison of NPCR, UACI between several encryption systems and the suggested method for gray images.

				Average
Suggested	NPCR	99.6167	99.5700	99.5934
	UACI	33.3253	33.2854	33.3054
Li et al. [14]	NPCR	99.6307	99.5850	99.6129
	UACI	33.4534	33.5582	33.5640
Enayatifar et al. [15]	NPCR	99.1051	99.5193	99.0406
	UACI	33.2517	33.5851	33.2617
Hosny et al. [16]	NPCR	99.5941	99.6246	99.6073
	UACI	33.4610	33.4226	33.4370

## 7 Conclusions

In this research work, an encryption technique is developed for both color and gray images. We create a matrix by dividing the original color image's RGB channels. Then, we adjust the pixel placements and values according to the 6D hyperchaotic map and symmetric matrix respectively. Using a 6D hyperchaotic map, the confusion stage is utilized to generate random sequences for the cipher algorithm, and then sequences $x_{1},x_{2},x_{6}$ are selected to modify the pixel location of plain pictures for each RGB channel. Each subblock of the scrambled picture has a unique pixel value in the diffusion operation, which we have transformed using the symmetric matrix for each RGB channel.

Incredibly safe image encryption and decryption algorithm are provided by control parameters of the 6D hyperchaotic map. This research developed an encryption technique with secret key that is 128 bits long, providing it a 2^128^ large key space. In summary, Fig. 6 represents the histogram analysis represents that the pixel's distribution is uniform. Figure 7, Figure 8, Figure 9, Figure 10 and Table 1, Table 2 explain that the correlation coefficient in the adjacent pixels is extremely less. Table 3, Table 4 show the average entropy values for color and grayscale images are 7.9992 and 7.9973, respectively. For color images, NPCR and UACI are 99.5956 and 33.4061, correspondingly, in Table 5 and for grayscale images, NPCR and UACI are 99.5934 and 33.3054, subsequently, in Table 6. The results offers a uniform histogram distribution for the encoded image. For entropy measurement, and is very near to the optimum entropy value, the encoded picture has a huge level of capriciousness, and the adjacent pixel correlation coefficient has drastically down in all three directions. According to the analysis's findings, we can say that the suggested scheme offered a decent degree of protection, enabling it to keep hackers out of colored and grayscale photographs on public platforms.

## Nomenclature

$\tau_{1},\tau_{2},\tau_{3},\tau_{4},\tau_{5}$: Constants of 6D hyperchaotic system
H(g): Information Entropy
*LE*: Lyapunov Exponent
NPCR: Number of Pixel Change Rate
$r_{s,t}$: Correlation Coefficient
UACI: Unified Average Changing Intensity
$x_{1},x_{2},x_{3},x_{4},x_{5},x_{6}$: Variables of 6D hyperchaotic system

## Code availability

Not applicable

## Funding

No specific external funding is available for this work.

## CRediT authorship contribution statement

**Anju Panwar:** Writing – review & editing, Methodology, Investigation. **Geeta Biban:** Writing – original draft, Resources, Investigation. **Renu Chugh:** Visualization, Validation, Supervision. **Asifa Tassaddiq:** Methodology, Conceptualization. **Rabab Alharbi:** Validation, Formal analysis, Data curation.

## Declaration of Competing Interest

We, the authors of the paper titled “An Efficient Image Encryption Model Based on 6D Hyperchaotic System and Symmetric Matrix For Color and Gray Images”, hereby declare that we have no financial or non-financial interests that could be perceived as influencing the objectivity or integrity of the work presented. We affirm that we have not received any external funding that may have biased the outcomes or conclusions of the paper. We confirm that the ideas, concepts, and intellectual property presented in the paper are original and do not infringe upon the rights of any third party. We acknowledge our responsibility to promptly disclose any actual or perceived conflicts of interest that may arise during the publication process. We understand that providing this declaration ensures transparency and upholds the credibility of our work.

## Data Availability

No new data was generated for this research.

## References

[1] Hosny K.M., Kamal S.T., Darwish M.M. (2022). A color image encryption technique using block scrambling and chaos. Multimed. Tools Appl..

[2] Teng L., Wang X., Xian Y. (2022). Image encryption algorithm based on a 2D-CLSS hyperchaotic map using simultaneous permutation and diffusion. Inf. Sci..

[3] Erkan U., Toktas A., Lai Q. (2023). 2D hyperchaotic system based on Schaffer function for image encryption. Expert Syst. Appl..

[4] Zhang Z., Tang J., Zhang F., Ni H., Chen J., Huang Z. (2022). Color image encryption using 2d sine-cosine coupling map. IEEE Access.

[5] Jasra B., Moon A.H. (2022). Color image encryption and authentication using dynamic DNA encoding and hyperchaotic system. Expert Syst. Appl..

[6] Wang S., Peng Q., Du B. (2022). Chaotic color image encryption based on 4D chaotic maps and DNA sequence. Opt. Laser Technol..

[7] Nazir H., Bajwa I.S., Abdullah S., Kazmi R., Samiullah M. (2022). A color image encryption scheme combining hyperchaos and genetic codes. IEEE Access.

[8] Yan S., Li L., Gu B., Cui Y., Wang J., Song J. (2023). Design of hyperchaotic system based on multi-scroll and its encryption algorithm in color image. Integration.

[9] Li X., Zeng J., Ding Q., Fan C. (2022). A novel color image encryption algorithm based on 5-D hyperchaotic system and DNA sequence. Entropy.

[10] Qiu H., Xu X., Jiang Z., Sun K., Xiao C. (2022). A color image encryption algorithm based on hyperchaotic map and Rubik's Cube scrambling. Nonlinear Dyn..

[11] Mir U.H., Singh D., Lone P.N. (2022). Color image encryption using RSA cryptosystem with a chaotic map in Hartley domain. Inf. Secur. J..

[12] Wang X., Su Y., Luo C., Nian F., Teng L. (2022). Color image encryption algorithm based on hyperchaotic system and improved quantum revolving gate. Multimed. Tools Appl..

[13] Kumar K., Roy S., Rawat U., Malhotra S. (2022). IEHC: an efficient image encryption technique using hybrid chaotic map. Chaos Solitons Fractals.

[14] Li C., Luo G., Qin K., Li C. (2017). An image encryption scheme based on chaotic tent map. Nonlinear Dyn..

[15] Enayatifar R., Abdullah A.H., Isnin I.F., Altameem A., Lee M. (2017). Image encryption using a synchronous permutation-diffusion technique. Opt. Lasers Eng..

[16] Hosny K.M., Kamal S.T., Darwish M.M., Papakostas G.A. (2021). New image encryption algorithm using hyperchaotic system and Fibonacci q-matrix. Electronics.

[17] Chugh R., Batra C., Biban G., Ahuja A. (2022). New four step iteration process for approximating fixed point of contraction mappings. J. MESA.

[18] Biban G., Panwar A. (2021). Data analysis of COVID-19 pandemic: a mathematical approach. J. Math. Comput. Sci..

[19] Gonzalez R.C. (2009).

[20] Gonzalez R.C., Eddins S.L., Woods R.E. (2004).

[21] Biban G., Chugh R., Panwar A. (2023). Image encryption based on 8D hyperchaotic system using Fibonacci Q-Matrix. Chaos Solitons Fractals.

[22] Chugh R., Batra C., Biban G., Ahuja A. (2022). New four step iteration process for approximating fixed point of contraction mappings. Math. Eng. Sci. Aerosp..

[23] Abbas S., Nazar M., Nisa Z.U., Amjad M., Din S.M.E., Alanzi A.M. (2022). Heat and mass transfer analysis of MHD Jeffrey fluid over a vertical plate with CPC fractional derivative. Symmetry.

[24] Abbas S., Gilani S.F.F., Nazar M., Fatima M., Ahmad M., Nisa Z.U. (2023). Bio-convection flow of fractionalized second grade fluid through a vertical channel with Fourier's and Fick's laws. Mod. Phys. Lett. B.

[25] Abbas S., Ahmad M., Nazar M., Amjad M., Ali H., Jan A.Z. (2023). Heat and mass transfer through a vertical channel for the Brinkman fluid using Prabhakar fractional derivative. Appl. Therm. Eng..

[26] Abbas S., Nisa Z.U., Nazar M., Amjad M., Ali H., Jan A.Z. (2024). Application of heat and mass transfer to convective flow of Casson fluids in a microchannel with Caputo–Fabrizio derivative approach. Arab. J. Sci. Eng..

[27] Abbas S., Ahmad M., Nazar M., Ahmad Z., Amjad M., Garalleh H.A., Jan A.Z. (2024). Soret effect on MHD Casson fluid over an accelerated plate with the help of constant proportional Caputo fractional derivative. ACS Omega.

[28] Abbas S., Ahmad M., Rahimzai A.A., Nazar M., Khan I. (2023).

[29] Arqub O.A., Abo-Hammour Z. (2014). Numerical solution of systems of second-order boundary value problems using continuous genetic algorithm. Inf. Sci..

[30] Abo-Hammour Z., Abu Arqub O., Momani S., Shawagfeh N. (2014). Optimization solution of Troesch's and Bratu's problems of ordinary type using novel continuous genetic algorithm. Discrete Dyn. Nat. Soc..

[31] Benkouider K., Bouden T., Yalcin M.E., Vaidyanathan S. (2020). A new family of 5D, 6D, 7D and 8D hyperchaotic systems from the 4D hyperchaotic Vaidyanathan system, the dynamic analysis of the 8D hyperchaotic system with six positive Lyapunov exponents and an application to secure communication design. Int. J. Model. Identif. Control.

[32] Bunch J.R., Kaufman L., Parlett B.N. (1976). Decomposition of a symmetric matrix. Numer. Math..

[33] Shannon C.E. (1951). Prediction and entropy of printed English. Bell Syst. Tech. J..

